# Phagocytosis of a PFOB-Nanoemulsion for ^19^F Magnetic Resonance Imaging: First Results in Monocytes of Patients with Stable Coronary Artery Disease and ST-Elevation Myocardial Infarction

**DOI:** 10.3390/molecules24112058

**Published:** 2019-05-30

**Authors:** Fabian Nienhaus, Denise Colley, Annika Jahn, Susanne Pfeiler, Vera Flocke, Sebastian Temme, Malte Kelm, Norbert Gerdes, Ulrich Flögel, Florian Bönner

**Affiliations:** 1 Cardiovascular Research Laboratory, Division of Cardiology, Pulmonology and Vascular Medicine, Medical Faculty, Heinrich-Heine-University, Moorenstrasse 5, 40225 Düsseldorf, Germany; fabiantheodor.nienhaus@med.uni-duesseldorf.de (F.N.); d.colley@gmx.de (D.C.); annika.jahn@med.uni-duesseldorf.de (A.J.); susanne.pfeiler@med.uni-duesseldorf.de (S.P.); malte.kelm@med.uni-duesseldorf.de (M.K.); norbert.gerdes@med.uni-duesseldorf.de (N.G.); 2 Experimental Cardiovascular Imaging, Department of Molecular Cardiology, Medical Faculty, Heinrich-Heine-University Düsseldorf, Moorenstrasse 5, 40225 Düsseldorf, Germany; flocke@uni-duesseldorf.de (V.F.); sebastian.temme@uni-duesseldorf.de (S.T.); floegel@uni-duesseldorf.de (U.F.); 3 CARID, Cardiovascular Research Institute Düsseldorf, Medical Faculty, Heinrich-Heine-University, Moorenstrasse 5, 40225 Düsseldorf, Germany

**Keywords:** ^19^F MRI, phagocytosis, monocytes, perfluorocarbons, perfluorooctyl bromide, PFOB, STEMI, inflammation

## Abstract

Fluorine-19 magnetic resonance imaging (^19^F MRI) with intravenously applied perfluorooctyl bromide-nanoemulsions (PFOB-NE) has proven its feasibility to visualize inflammatory processes in experimental disease models. This approach is based on the properties of monocytes/macrophages to ingest PFOB-NE particles enabling specific cell tracking in vivo. However, information on safety (cellular function and viability), mechanism of ingestion and impact of specific disease environment on PFOB-NE uptake is lacking. This information is, however, crucial for the interpretation of ^19^F MRI signals and a possible translation to clinical application. To address these issues, whole blood samples were collected from patients with acute ST-elevation myocardial infarction (STEMI), stable coronary artery disease (SCAD) and healthy volunteers. Samples were exposed to fluorescently-labeled PFOB-NE and particle uptake, cell viability and migration activity was evaluated by flow cytometry and MRI. We were able to show that PFOB-NE is ingested by human monocytes in a time- and subset-dependent manner via active phagocytosis. Monocyte function (migration, phagocytosis) and viability was maintained after PFOB-NE uptake. Monocytes of STEMI and SCAD patients did not differ in their maximal PFOB-NE uptake compared to healthy controls. In sum, our study provides further evidence for a safe translation of PFOB-NE for imaging purposes in humans.

## 1. Introduction

Non-invasive visualization of macrophages is of paramount interest in various clinical inflammatory diseases, since those cells impact on prognosis. Especially, cardiovascular inflammation with respect to atherosclerosis or myocardial healing after acute myocardial infarction (AMI) is centrally orchestrated by monocytes and macrophages [1,2]. Thus, there is a clinical need for a direct and cell specific labelling of monocytes and macrophages in cardiovascular disease. Fluorine-19 magnetic resonance imaging (^19^F MRI) with intravenously applied Perfluorocarbon-nanoemulsions (PFC-NE) has proven its feasibility to visualize inflammatory processes in a wide range of experimental disease models (e.g., myocardial infarction, stroke, graft rejection, myocarditis) [3,4,5,6,7,8]. This imaging approach is based on the properties of monocytes/macrophages to ingest PFC-NE particles with high affinity, which allows a cell type-specific tracking in vivo [3]. The specificity of this approach can even be increased by functionalizing the respective nanoemulsions using immune-targeting strategies [9]. Since there is no natural ^19^F background in the mammalian body, the ^19^F signal is directly proportional to the amount of ingested particles, making this method directly quantifiable. Furthermore, perfluorocarbon (PFC) compounds are considered biologically inert providing optimal characteristics for clinical application. 

Among different PFC compounds, perfluoro-15-crown-5 ether (PFCE) and perfluorooctyl bromide (PFOB, also referred to as Perflubron) are the most widely used agents in ^19^F MRI studies [3,4,5,6,7,8,10,11]. Although PFCE provides greater sensitivity due to its 20 equivalent ^19^F atoms, it accumulates in liver and spleen for months and is therefore not suitable for translation to human use [3]. In contrast, PFOB has a shorter half-life of 12 days and is exhaled through the lung [12,13]. Moreover, PFOB-NE has already undergone clinical trials making use of its oxygen transport characteristics in patients expecting hemorrhagic surgery [14]. Furthermore, PFOB is currently used in ongoing clinical trials to evaluate the safety of PFOB in partial liquid ventilation in neonates with severe bronchopulmonary dysplasia as well as in the treatment of cystic fibrosis. 

In translational imaging studies, ^19^F MRI using PFOB-NE was successfully transferred from experimental MR scanners to clinical scanners and preclinical large animal models: Here, myocardial inflammation was detected following acute myocardial infarction (AMI) in explanted hearts [10]. Recently, these proceedings were extended to an in vivo setting within a reasonable imaging time (<20 min) [11].

Potential clinical translation for imaging purposes crucially requires information on cell survival and cell function after phagocytosis of PFOB-NE, as monocytes/macrophages play a pivotal role in inflammatory processes (e.g., in infarct healing or infectious defense). Furthermore, for the interpretation of ^19^F images it is critical to know, whether underlying inflammatory diseases have an impact on the phagocytic activity of monocytes/macrophages as this could possibly change ^19^F signal intensity in different groups of patients.

Hence, this study aimed to gain further insight into the subset-specific uptake of PFOB-NE in peripheral human blood monocytes, the mechanism of ingestion and the impact of particle ingestion on cellular function like migration and phagocytosis. Furthermore, we determined whether inflammatory diseases like stable coronary artery disease (SCAD) or ST-elevation myocardial infarction (STEMI) have an influence on PFOB-NE uptake.

## 2. Results

### 2.1. Properties of the Applied PFOB-NE

**Figure 1:**
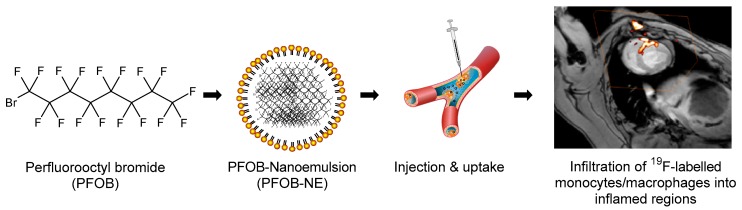
Workflow of perfluorooctyl bromide-nanoemulsions (PFOB-NE) application in pre-clinical animal models (modified from Flögel, et al. [3] and Jacoby, et al. [13]). An example of local inflammation after myocardial infarction as detected by ^19^F MRI in a large animal model at clinical field strength (3 Tesla) is shown on the right (Modified and reprinted from [11] with permission from Springer Nature, original copyright 2018).

Figure 1 sums up the basic workflow for PFOB-NE application in pre-clinical animal models. Briefly, PFOB is emulsified under high pressure (1000 bar) using a laboratory processor (Microfluidizer M-110P). The resulting PFOB-NE can then be injected intravenously and is predominantly taken up by monocytes/macrophages. Accumulation of PFOB-NE loaded cells in inflamed regions can then be visualized using ^19^F MRI as shown for local inflammation following acute myocardial infarction in pigs [11]. 

Table 1 shows basic properties of the PFOB-NE used for the following experiments. Particle size and zeta potential were measured using dynamic light scattering and presented as mean ± SD. Notably, mean particle size was 209 ± 17.4 nm and was therefore similar in size to those PFOB-NE particles used before in preclinical studies with pigs (200 ± 30 nm) [11]. 

### 2.2. PFOB-NE is Mainly Ingested by Human Monocytes

**Figure 2:**
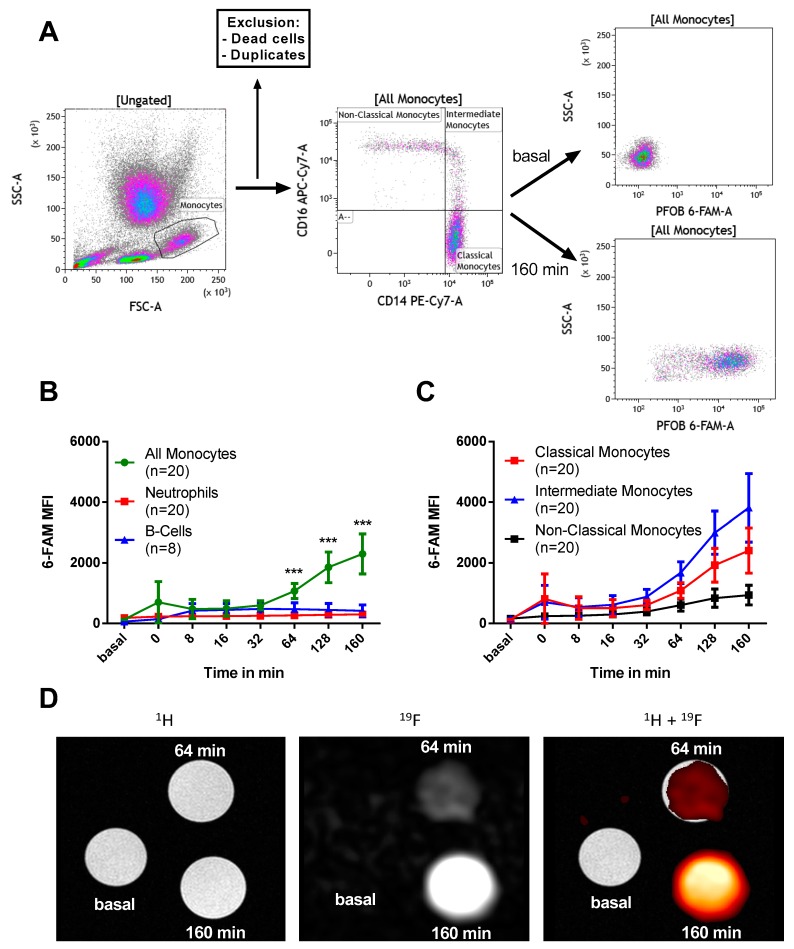
PFOB-NE is taken up by human leukocytes in a time- and species-dependent manner as measured by flow cytometry (**A** + **B** + **C**) and magnetic resonance imaging (**D**). (**A**) Representative gating strategy for monocytes is shown. Monocytes were identified by size and granularity (FSC and SSC). After exclusion of dead cells and cell duplicates monocyte subsets were identified by CD14 and CD16 staining. 6-FAM MFI was measured over time. (**B**) 6-FAM MFI measurements in monocytes, neutrophils and B cells and in (**C**) different monocyte subsets. (**D**) Shown are cross-sectional MR-images of tubes containing isolated monocytes at indicated time-points after PFOB-NE exposure. In ^1^H-MRI, all tubes are equivalently visible. In ^19^F MRI, there was a time-dependent signal increase. A merge of ^1^H and ^19^F MRI is shown on the right. For the sake of clarity, a hot iron color look-up table was applied to ^19^F MRI data.

Anti-coagulated whole blood samples were collected from patients with SCAD and co-incubated with 6-Carboxyfluorescein (6-FAM) labeled PFOB-NE. Particle uptake was measured by flow cytometry or ^19^F MRI. Figure 2A shows a representative example of our gating strategy for monocyte subsets via flow cytometry. For a representative example of the gating strategy for other leukocyte subtypes please see supplementary Figures S1–S3. Following 160 min of PFOB-NE co-incubation, a clear increase in median fluorescence intensity (MFI) was observed compared to non-PFOB-NE exposed monocytes.

Figure 2B,C show 6-FAM fluorescence intensity measurements in different leukocyte and monocyte subsets. Monocytes show an increased uptake of PFOB-NE in comparison to neutrophils and B cells starting at 64 min after exposure (Figure 2B). In contrast, Neutrophils and B cells show a small increase in MFI 8 min after PFOB-NE exposure and no further MFI increase afterwards. Within the different monocyte subsets, we detected that intermediate monocytes had the highest uptake of PFOB-NE while classical monocytes acquired a moderate signal (Figure 2C). Non-classical monocytes only showed a minor uptake. Starting at 64 min, MFI was significantly higher for intermediate monocytes compared to non-classical monocytes (*p* = 0.001). At 128 and 160 min there was a significant difference between all of the groups. 

Notably, those results could be reproduced using ^19^F MRI as second read out technology (Figure 2D). Whole blood samples were exposed to PFOB-NE and CD14^+^ monocytes were then isolated using magnetic cell separation. In cross-sectional ^1^H MRI all tubes are equivalently visible. In ^19^F MRI we observed a time-dependent signal increase with a 4-fold enhanced signal intensity compared to baseline measurements. 

### 2.3. PFOB-NE is Ingested by is Active Phagocytosis

**Figure 3:**
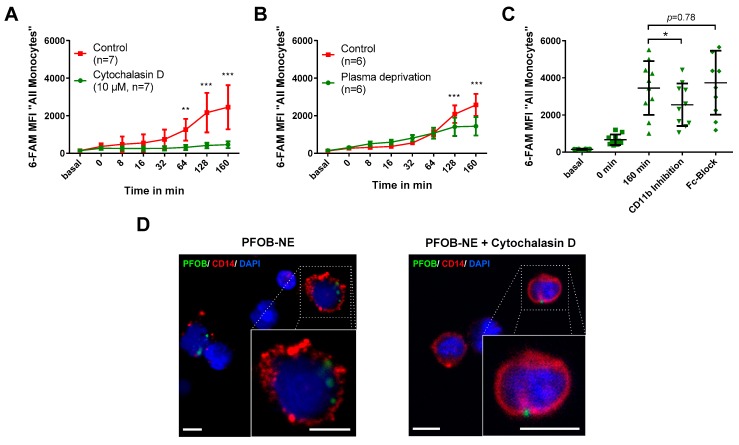
PFOB-NE is internalized by active phagocytosis. Whole blood samples were exposed to PFOB-NE (**A**) with or *w*/*o* use of Cytochalasin D (*n* = 7), (**B**) with or *w*/*o* plasma deprivation (*n* = 6) or (**C**) with or *w*/*o* CD11b- or Fc-inhibiton (*n* = 10) and analysed for cell-associated fluorescence by flow cytometry. (**D**) Confocal analysis of PFOB-NE internalization in CD14^+^ monocytes (red). Whole blood samples were pre-incubated with or *w*/*o* Cytochalasin D and subsequently exposed to PFOB-NE (green) for 128 min. Nuclei were counterstained with DAPI (blue). Small boxes indicate area magnified in large boxes. All scale bars, 5 µm.* *p* < 0.05, ** *p* < 0.01, *** *p* < 0.001.

To characterize the pathway of PFOB-NE uptake into human monocytes we used Cytochalasin D, a known inhibitor of actin polymerization [15,16]. When Cytochalasin D was added to the co-incubation of whole blood and PFOB-NE only a minor increase in monocyte MFI was observed over time. In contrast, the control group showed a significant increase of MFI compared to the Cytochalasin D group beginning at 64 min after PFOB-NE exposure (Figure 3A). Therefore, this experiment suggests a specific uptake of PFOB-NE via actin-dependent phagocytosis.

In the next step we tested whether an opsonization by plasma proteins is required for phagocytosis. Whole blood samples were centrifuged and blood plasma was replaced by phosphate buffered saline (PBS) (Figure 3B). Plasma deprivation still lead to a significant increase of monocyte MFI over time (*p* = 0.001). However, the control group using full blood showed a further increase in fluorescence beginning at 128 min after PFOB-NE exposure.

We further wanted to elucidate if complement (CD11b)- or Fc-receptors play a role in plasma-dependent phagocytosis as has been shown for other particles [17,18,19]. Therefore, we used a functional blocking CD11b antibody and an Fc-blocking antibody to study the impact of both receptors on phagocytosis of PFOB-NE (Figure 3C). A significant decrease of monocyte MFI could be observed using CD11b antibody while blocking of Fc-receptors had no significant effect on PFOB-NE particle uptake.

To prove that PFOB-NE is actually taken up into the cell and is not just attached to the cell surface, we used fluorescence microscopy (Figure 3D). Here we could confirm that 6-FAM-labeled PFOB-NE was internalized by CD14-positive cells at 128 min of incubation whereas in the presence of Cytochalasin D no internalized particles could be found.

### 2.4. Viability and Subset Reclassification after PFOB-Ingestion

**Figure 4:**
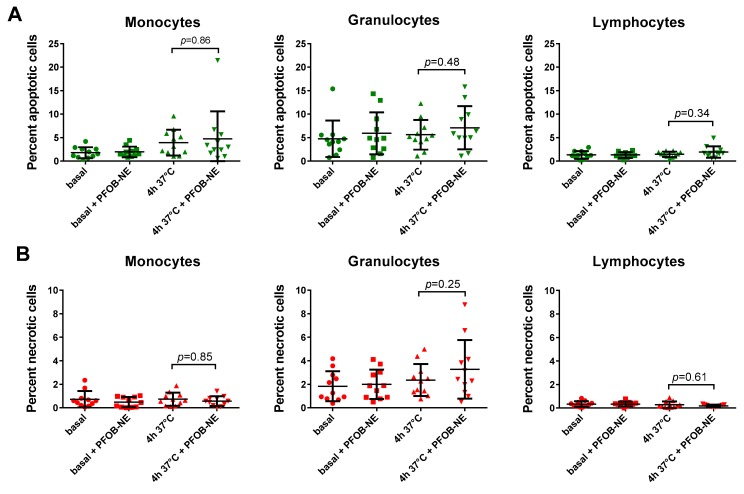
Leukocytes remain viable after PFOB-NE ingestion. The graphs show the percentage of (**A**) apoptotic and (**B**) necrotic monocytes, granulocytes and lymphocytes after 4 h of PFOB-NE exposure. Cell viability was discriminated via flow cytometry. No significant differences in the percentage of apoptotic or necrotic cells were observed after 4 h of PFOB-NE exposure (*n* = 11 each).

To test leukocyte viability after PFOB-NE exposure we used whole blood samples exposed to PFOB-NE for 4 hours and analyzed them by flow cytometry. For a representative example of our gating strategy please see supplementary Figure S4. The analysis revealed no significant difference in the percentage of apoptotic (Annexin V^+^, Zombie^−^) or necrotic (Annexin V^+^, Zombie^+^) cells in any of the indicated leukocyte subpopulations (Figure 4). 

**Figure 5:**
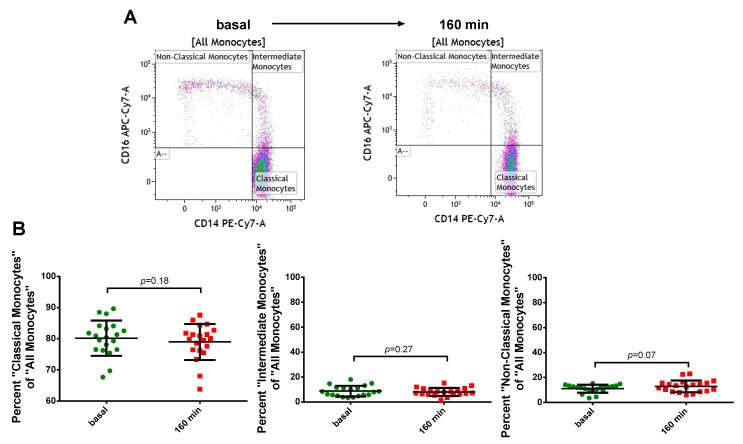
Monocytes keep their antigen classification after PFOB-NE ingestion. Whole blood samples were analyzed before and after exposure to PFOB-NE for 160 min. The percentage of different monocyte subsets of all monocytes was classified using flow cytometry. (**A**) Representative example and (**B**) quantitative analysis for monocyte subset classification.

Since monocytes might change their surface antigens upon inflammatory activation we investigated the effect of PFOB-NE ingestion upon surface antigen presentation of CD14 and CD16. CD14 mediates the lipopolysaccharide (LPS)-induced host protection against bacterial infections [20] and CD16 is an Fc-receptor involved in antibody-mediated phagocytosis [21]. Monocyte subgroup classification was assessed before and after 160 min of PFOB-NE exposure (Figure 5). No significant difference could be observed in the percentage of different monocyte subsets after PFOB-NE ingestion.

### 2.5. Monocyte Function after PFOB-NE Exposure

**Figure 6:**
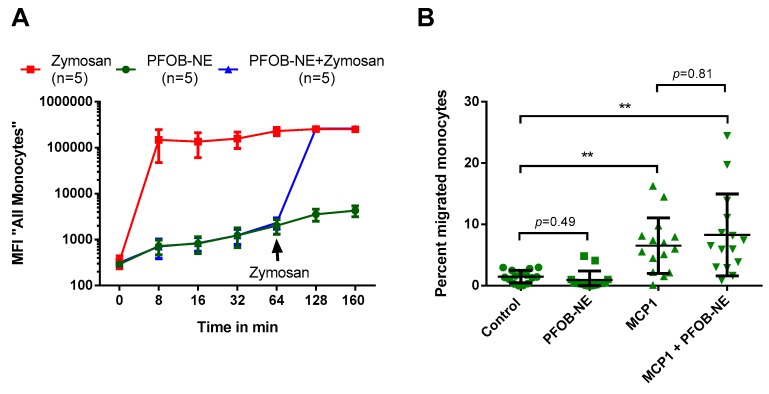
Monocytes remain functional after PFOB-NE ingestion. (**A**) Whole blood samples were either exposed to 6-FAM-labeled PFOB-NE or FITC-labeled Zymosan, a reference marker for phagocytosis. In a third group Zymosan was added 64 min after PFOB-NE exposure. Fluorescence uptake was monitored by flow cytometry. No difference in the amount of ingested Zymosan was observed between monocytes pre-stimulated or not pre-stimulated with PFOB-NE. (**B**) Monocyte migration was evaluated using a transwell migration assay with a pore size of 3 µm. Migration was stimulated by MCP1 (10 ng/mL). The percentage of migrated monocytes was evaluated using flow cytometry. Pre-stimulation with PFOB-NE for 160 min did not have an impact on the percentage of migrated monocytes. *n* = 15, ** *p* < 0.01.

Phagocytosis and migration are crucial functions of human monocytes. To investigate the effect of PFOB-NE ingestion on phagocytic ability of human monocytes, we performed a phagocytosis assay with co-use of 6-FAM-labeled PFOB-NE and Fluorescein isothiocyanate (FITC)-labeled Zymosan, which is a yeast particle known to be phagocytosed by different cells types including monocytes/macrophages [22]. It can therefore serve as a marker for phagocytosis. Whole blood samples were exposed to PFOB-NE or Zymosan (200 µg/mL) for the indicated time. In a third group Zymosan was added 64 min after PFOB-NE exposure. There was no significant difference between the phagocytic capacity of monocytes for Zymosan particles before or 64 min after PFOB-NE exposure (Figure 6A). 

To evaluate the impact of PFOB-NE challenge on monocyte migration we used a transwell migration assay. Whole blood samples were pre-exposed to PFOB-NE for 160 min or left untreated as control. Mononuclear cells were isolated and monocyte migration from the upper to the lower compartment was or was not stimulated by monocyte chemoattractant protein 1 (MCP1). True to form, there was a significant increase in the percentage of migrated monocytes stimulated with MCP1. However, PFOB-NE challenge did not have an impact on the percentage of migrated monocytes neither with nor without MCP1 stimulation (Figure 6B).

### 2.6. Association of Myocardial Infarction with Phagocytosis of PFOB-NE by Human Monocytes

In a last step, the effect of AMI on the phagocytic capacity of monocytes was tested. AMI induces a sterile inflammatory response syndrome and might impact on phagocytic properties of human monocytes, which could have an impact on ^19^F signals.

**Figure 7:**
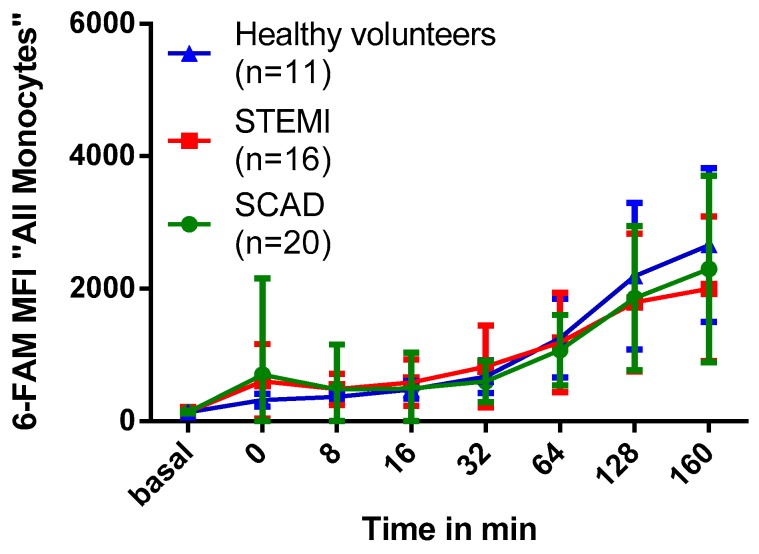
Impact of myocardial infarction on PFOB-NE phagocytosis. Whole blood samples of patients with STEMI, SCAD or healthy volunteers were exposed to PFOB-NE for different periods of time. MFI in the 6-FAM-channel as a parameter for the amount of ingested particles was evaluated by flow cytometry. No significant differences in PFOB-NE uptake of human monocytes could be observed between patients with STEMI, SCAD and healthy volunteers.

To this end, we compared monocyte phagocytic capacity of patients at day three after STEMI to aged-matched patients with SCAD and young healthy volunteers. For patients characteristics see supplementary Table S2. As shown in Figure 7, no significant difference could be observed in monocyte MFI in the 6-FAM-channel of patients with STEMI, SCAD and healthy volunteers (*p* = 0.96). Furthermore, there was no correlation between the maximum MFI in the 6-FAM-channel and markers for infarct size (maximum creatine kinase level, MR-measured infarct size) or co-morbidities (see supplementary Table S3). 

## 3. Discussion

Our study shows that PFOB-NE co-incubated with human whole blood is 1) preferentially taken up by monocytes (Figure 2); 2) phagocytosed via CD11b-dependent mechanisms (Figure 3), thereby not inducing apoptosis and necrosis (Figure 4); 3) does not interfere with migratory function or phagocytic capacity of monocytes (Figure 6); and 4) is not significantly different between volunteers and patients with STEMI or SCAD (Figure 7).

### 3.1. Cell Type and Subset Specific PFOB-NE Uptake

Experimental approaches in mice suggest that after intravenous injection PFOB-NE is mainly ingested by monocytes and to a much lesser extent by B cells and neutrophils [3,6]. These findings are now reproduced in human peripheral blood cells. Moreover, we could elucidate a monocyte subset specific phagocytic capacity: PFOB-NE is preferentially taken up by intermediate monocytes followed by classical and to a much lesser extend by non-classical monocytes. All other leukocytes internalize PFOB-NE to a much lesser extent (Figure 2). These findings are in line with other studies on phagocytic properties of monocyte subsets [23]. Although intermediate monocytes show the highest phagocytic potential, the main fluorescence signal derives from classical monocytes as their group represents about 80–85% of all monocytes. In terms of subset function, classical and intermediate monocytes are thought to have a more inflammatory phenotype while non-classical monocytes are rather patrolling and responsible for tissue regeneration [24,25]. Interestingly, increasing levels of circulating classical and intermediate monocytes can be found after STEMI with peak levels from day 1 up to day 3 after infarction [26,27]. High levels of intermediate monocytes even correlate with major adverse cardiac events (MACE) [26]. This highlights the importance of the two subsets for the acute inflammatory reaction and outcome of patients after myocardial infarction and makes them a highly relevant target for in vivo cell tracking. By in vitro imaging of sorted monocytes we could confirm that the differences in PFOB-NE uptake cannot only be visualized by flow cytometry but also by ^19^F MRI (Figure 2). 

### 3.2. Monocyte Function, Viability and Subgroup Distribution after PFOB-NE Ingestion

Monocytes play a pivotal role in sterile or infective inflammation [2,28]. Therefore, changes in monocyte viability, function or subset affiliation due to cell labeling could possibly lead to a disturbed inflammatory response resulting in immune dysfunction or inefficient infarct healing. Our data suggests that labeling monocytes with PFOB-NE for detection by ^19^F MRI does not cause any changes in monocyte viability (Figure 4) or subgroup distribution (Figure 5). Furthermore, crucial monocyte functions like phagocytosis and migration are preserved (Figure 6). These findings are not surprising as Perfluorocarbons are known to be one of the most inert substances [12,29,30]. They are biologically stable and cannot be metabolized. In agreement to our data, loading of murine dendritic cells with perfluoro-15-crown-5 ether (PFCE), another PFC-compound, had almost no effect on cellular integrity, proliferation, phenotype and metabolism [31]. Furthermore, ^19^F MRI confirms the infiltrating kinetics of in vivo labeled monocytes in various inflammatory disease models showing a proper healing pattern at long term observation thereby supporting our finding of a preserved cell function [3,4,5,6,7,8]. However, we are the first to study the effects of a PFOB-containing nanoemulsion on human peripheral blood monocytes. As ^19^F MRI with PFOB-NE has already proven its ability to visualize inflammatory processes in pre-clinical large animal models [10,11], these findings are of translational significance.

However, our data is limited to the relatively short observation period after PFOB-NE exposure. As our study is based on flow-cytometry assays, longer observation periods could lead to spontaneous cellular apoptosis and inadequate sample properties for flow cytometry analysis. But as our phagocytosis assay uses a whole blood analysis, cells are evaluated in a more natural environment than in cell culture studies.

### 3.3. Mechanism of PFOB-NE Ingestion

Another goal of this study was to gain deeper insights into the mechanism of PFOB-NE ingestion. As PFC-NE are mainly ingested by monocytes/macrophages and not by T-cells lacking phagocytic receptors, it has been hypothesized that receptor-mediated phagocytosis is the main mechanism of uptake [29]. Our data show that Cytochalasin D, a potent inhibitor of actin polymerization, nearly completely inhibited PFOB-NE uptake (Figure 3). It is known that actin polymerization and cytoskeletal movement are crucial processes for phagocytosis and have variable effects on other mechanisms of ingestion like clathrin-dependent or -independent endocytosis [32,33]. However, endocytosis occurs in every cell while phagocytosis is restricted to distinct cell lines like e.g. macrophages, monocytes and neutrophils [34]. As PFOB-NE is mostly taken up by monocytes/macrophages it suggests a specific phagocytic mechanism rather than an unspecific uptake by endocytosis. Furthermore, Cytochalasin D inhibits the ingestion of PFOB-NE particles but not the binding to cell surface proteins. Therefore, our Cytochalasin D studies provide evidence that the main signal of our assay derives from ingested PFOB-NE particles rather than from particles bound to the cell surface. This is supported by our confocal microscopy studies showing an intracellular location of PFOB-NE in contrast to a Cytochalasin D-preparation (Figure 3). Our Cytochalasin D studies further suggest that active phagocytosis starts about 32–64 min after PFOB-NE exposure. Before this time no significant differences could be found between Cytochalasin D treated and not treated monocytes. However, 0 to 32 min after PFOB-NE exposure MFI increases slightly which might reflect on the cell surface bond of PFOB-NE particles or other forms of PFOB-NE ingestion, like clathrin-mediated endocytosis.

Phagocytosis is a receptor-mediated event. Some ligands directly bind to a specific receptor while others require the opsonization of plasma proteins [35]. Our data show a significantly lower PFOB-NE uptake in the absence of blood plasma after 128 min and 160 min of PFOB-NE exposure (Figure 3). Furthermore, inhibition of complement receptor CD11b but not inhibition of immunoglobulin Fc-receptors was able to reduce PFOB-NE uptake (Figure 3). Complement receptor CD11b and receptors for the Fc-part of antibodies are widely known to be involved in phagocytic processes [17,18,19]. CD11b is involved in complement-mediated phagocytosis in monocytes [19]. Therefore, opsonization via the complement system seems to play a crucial role for PFOB-NE phagocytosis. As CD11b receptor blockage not completely inhibited phagocytosis additional receptors probably contribute to PFOB-NE phagocytosis. Promising candidates could be, for example, scavenger receptors or other complement receptors like CR1 or CR4 [35]. However, absence of blood plasma did not completely inhibit PFOB-NE uptake either. Thus, opsonization-independent pathways also seem to play a role in PFOB-NE ingestion. Whether the opsonization-independent uptake is due to phagocytosis or other mechanisms of endocytosis remains unknown and could be subject for future studies.

Taken together, our data suggest that the main mechanism of PFOB-NE ingestion is active phagocytosis that is partly dependent on opsonization via the complement system.

### 3.4. Impact of Myocardial Infarction on Monocyte Phagocytic Activity

^19^F signal intensity as measured by MRI can be influenced by the absolute monocyte count in an image-voxel but also by the median particle uptake of every single monocyte. Therefore, underlying diseases with influence on the phagocytic activity of monocytes could have an impact on ^19^F signal intensity and counterbalance cell count dependent ^19^F signal. Information on the impact of different inflammatory diseases on the phagocytic activity of peripheral human blood monocytes is sparse. In our study we evaluated the impact of acute STEMI on monocyte phagocytic ability. Here, no significant difference in PFOB-NE particle uptake could be observed between patients three days after STEMI, aged-matched patients with SCAD and young healthy volunteers (Figure 7). Furthermore, we could not find any correlation between several patient characteristics and maximal MFI as measured by flow cytometry. 

Data on monocyte phagocytic activity after acute myocardial infarction in humans is sparse. *Djurdjevic* et al. could show that the ability of peripheral blood mononuclear cells to ingest yeast particles increased at day 0 and 1 but not day 7 after acute myocardial infarction [36]. In line with this, monocyte subsets show highest blood levels of classical and intermediate monocytes at early time points after STEMI (day 2 and 3) with normalization at day 7 [26]. As classical and intermediate monocytes show higher phagocytic ability of PFOB-NE compared to non-classical monocytes, day 2 or 3 after STEMI could be the ideal time point for clinical application of PFOB-NE in STEMI patients. Furthermore, application of PFOB-NE at day 3 after myocardial infarction leads to significant ^19^F signals in the myocardium in large animals [10,11]. 

### 3.5. Future Prospects

In our study we analyzed patients with STEMI and SCAD and subsequent sterile inflammatory response. Future studies are needed to investigate if infective inflammatory diseases have a different impact on PFOB-NE uptake in human monocytes. 

## 4. Materials and Methods 

### 4.1. Collection of Blood Samples and Study Population

Heparinized whole blood samples were collected from patients during hospitalization in the department of cardiology of the University hospital of Düsseldorf. Eligible criteria were age > 40 years, informed consent and the presence of either angiographically proven SCAD or day 3 after reperfused STEMI. Furthermore, young volunteers without pre-existing conditions were included. For detailed information on inclusion and exclusion criteria and patient characteristics please see supplementary Tables S1 and S2. A total of 16 patients with STEMI, 20 patients with SCAD and 11 healthy volunteers were included. The study conformed to the Declaration of Helsinki and was approved by the University of Düsseldorf Ethics Committee. Informed consent was obtained from all patients.

### 4.2. Preparation of PFOB-NE

PFOB-NE was prepared as described elsewhere [3,10,13]. Briefly, purified egg lecithin (E 80 S, 4% *wt*/*wt*, Lipoid GmbH, Ludwigshafen, Germany) was dispersed in phosphate buffer (10 mM, pH 7.4) with 2.5% Glycerol by magnetic stirring at room temperature. Then 80.5 g PFOB (AtoChem, Puteaux, France) and for fluorescence measurements 5 mg 1,2-dioleoyl-sn-glycerol-3- phosphoethanolamine-*N*-(carboxyfluorescein) (6-FAM) (Avanti Polar Lipids, Inc., Alabaster, AL, USA) were added. Emulsions were stabilized by adding a semifluorinated alkane, which is a mixed fluorocarbon/hydrocarbon diblock compound (C_6_F_13_C_10_H_21_, *F*6*H*10) equimolar to the E 80 S lipid. Afterwards the dispersion was pretreated with a high-performance disperser (T18 basic ULTRA TURRAX, IKA Werke GmbH & CO. KG, Staufen, Germany) at 14,000 rpm for 2 min. This pre-emulsion was further emulsified by high-pressure/shear homogenization (1000 bar, 30 min) using a microfluidizer (Microfluidizer M-110P, Microfluidics Corp., Newton, MA, USA).

### 4.3. In Vitro Uptake of PFOB-NE by Human Leukocytes

Heparinized human whole blood samples were incubated with 6-FAM-labeled PFOB-NE at a dilution of 1:20 at 37 °C under constant shaking. At defined time points (basal, 0, 8, 16, 32, 64, 128, 160 min after PFOB-NE exposure) 200 µL of whole blood was taken, placed on ice and lysed three times for 15 min using an isotonic ammonium chloride buffer (NH_4_Cl 8.29 mg/mL, NaHCO_3_ 1 mg/mL, EDTA 0.0375 mg/mL in ddH_2_O). Afterwards leukocytes were re-suspended in FACS-buffer (2% FCS, 2 mmol/L EDTA in PBS) and pre-incubated with Fc-blocking reagent (1:100, Miltenyi Biotec GmbH, Bergisch Gladbach, Germany) for 15 min. Leukocytes were washed again and stained with antibodies specific for CD14 (clone: M5E2, source: mouse), CD16 (clone: 3G8, source: mouse), CD3 (clone: UCHT1, source: mouse) and/or CD19 (clone: HIB19, source: mouse) (BioLegend BioLegend, San Diego, CA, USA) for 15 min in the dark. After another washing step the cells were diluted with 250 µL of FACS-buffer containing 4′,6-Diamidin-2-phenylindol (DAPI) (1:250). For analysis of particle uptake, flow cytometry was performed on *FACSVerse* (BD Biosciences, San Jose, CA, USA) equipped with three lasers and capable of detecting up to eight colors. Data were acquired with *FACSuite* (BD Biosciences) and analyzed with *Kaluza* (Beckman Coulter, Inc., Brea, CA, USA). In our gating strategy monocytes, granulocytes or lymphocytes were located in FCS and SSC, cell duplicates and dead cells were excluded by DAPI staining. Monocyte subsets were discriminated by the expression of CD14 and CD16, granulocytes by the expression of CD16 and lymphocytes by the expression of CD3 and CD19. Median fluorescence intensity (MFI) in the 6-FAM-channel (absorption wavelength 495 nm, emission wavelength 517 nm) was measured as a parameter for the amount of ingested PFOB-NE using *Kaluza* (Beckman Coulter). For representative examples of the different gating strategies please see supplementary Figures S1–S3. FITC-labeled Zymosan (Thermo Fisher Scientific, Waltham, MA USA) was used as a reference marker for phagocytosis in a concentration of 200 µg/mL.

For mechanistic studies Cytochalasin D (Sigma Aldrich, St. Louis, MO, USA) in a final concentration of 10 µM, LEAF purified anti-human CD11b antibody (BioLegend, clone: ICRF44, source: mouse) in a concentration of 40 µg/mL or Fc-Blocking reagent (anti CD16/32) (Miltenyi) in a dilution of 1:25 were added to a whole blood sample 30 min before PFOB-NE exposure. Afterwards a phagocytosis assay was performed as described above. In further mechanistic studies whole blood samples were centrifuged (500 RCF, 7 min) and blood plasma was replaced by the same volume of PBS. Cells were washed 3 times with PBS and a phagocytosis assay was performed as described above under use of PBS instead of FACS-buffer.

### 4.4. Determination of Apoptotic and Necrotic Leukocytes after PFOB-NE Ingestion

For determination of apoptotic and necrotic cells, an Annexin V/Zombie assay (BioLegend) was used. Heparinized human whole blood samples were incubated with 6-FAM-labeled PFOB-NE at a dilution of 1:20 at 37 °C under constant shaking for 4 h. 200 µL of whole blood was taken, placed on ice and lysed 3 times for 15 min. Afterwards, leukocytes were re-suspended in PBS and incubated with Zombie Aqua Fixable Dye (BioLegend, 1:100) for 15 min. Cells were then washed with FACS-buffer (2% FCS, 2 mmol/L EDTA in PBS), re-suspended in Annexin V binding buffer and stained with APC-labeled Annexin V (BioLegend, 1:20) for 15 min. Flow cytometry was performed as described above. In our gating strategy neutrophils, monocytes or lymphocytes were located in the FSC and SSC, cell duplicates and dead cells were excluded and necrotic, apoptotic and living cells were discriminated by Annexin V and Zombie staining. A representative example of the gating strategy is shown in supplementary Figure S4.

### 4.5. ^19^F MRI of Isolated Human Monocytes after PFOB-NE Ingestion

For magnetic separation of CD14-positive monocytes we used StraightFrom Whole Blood CD 14 MicroBeads (Miltenyi). Heparinized human whole blood was incubated with 6-FAM-labeled PFOB-NE at a dilution of 1:20 at 37 °C under constant shaking for 64 or 160 min. StraightFrom Whole Blood CD14 MicroBeads were added to the sample and incubated for 15 min at 4 °C. Magnetic-separation of CD14-positiv cells was performed directly from whole blood by using the QuadroMACS Separator (Miltenyi) and Whole blood column kit (Miltenyi). Isolated CD14-positive cells were collected in the Whole blood elution buffer (Miltenyi). To remove remaining iron from the cell surface that disturbs MR imaging, CD14-positive cells were lysed with RIPA-buffer for 15 min (100 µL RIPA-buffer per 1 × 10^6^ cells). Magnetic-separation was performed again using the QuadroMACS Separator (Miltenyi) and LS columns (Miltenyi). The magnetically negative lysate was collected and ^1^H- as well as ^19^F MRI was performed. Data were recorded on a Bruker DRX 9.4 Tesla Wide Bore (89 mm) NMR spectrometer (Bruker, Billerica, MA, USA) operating at frequencies of 400.13 MHz for ^1^H and 376.46 MHz for ^19^F measurements. A Bruker microimaging unit (Mini 0.5) equipped with an actively shielded 57 mm gradient set was used and images were obtained from a 30 mm birdcage resonator tunable to ^1^H and ^19^F. After acquisition of the cross-sectional morphological ^1^H images, the resonator was tuned to ^19^F and anatomically matching ^19^F images were recorded. For better visibility in the merged picture, a hot iron color look-up table was applied to ^19^F MRI data showing equal signal intensity as the black and white scale.

### 4.6. Migration Assay

In our migration studies we used a commercial migration assay in a 12-well format with a pore size of 3 µm (Thin Cert^TM^, Greiner Bio-One International GmbH, Kremsmünster, Austria). Heparinized whole blood samples were incubated with 6-FAM-labeled PFOB-NE at a dilution of 1:20 at 37 °C under constant shaking for 160 min. Afterwards whole blood was transferred to BD Vacutainer® CPT^TM^ Mononuclear cell preparation tubes. Mononuclear cells were isolated by centrifugation (1500 RCF for 20 min) and transferred to centrifuge tubes. Cells were washed, incubated in Dulbecco′s Modified Eagle′s Medium (DMEM) with 10% FCS and counted via flow cytometry using CountBright Absolute counting beads (Thermo Fisher Scientific). 500,000 monocytes were transferred to the upper compartment of a Thin Cert™ well. In the lower compartment Monocyte chemoattractant protein 1 (MCP-1) was added to 2 mL DMEM with 10% FCS in a concentration of 10 ng/mL to stimulate monocyte migration. After an incubation period of 3 h at 37 °C, medium of the lower compartment was taken without touching the upper compartment. Migrated cells were stained with antibodies for CD14 and CD16 epitope (BioLegend) and counted via flow cytometry using CountBright Absolute counting beads (Thermo Fisher Scientific).

### 4.7. Imaging in Vitro Uptake of PFOB-NE in CD14^+^ Cells

Heparinized human whole blood samples were incubated with 6-FAM-labeled PFOB-NE with or without presence of Cytochalasin D (10 µM) at a dilution of 1:20 at 37 °C under constant shaking. After 128 min a probe of 500 µL of whole blood was taken, placed on ice and lysed three times for 15 min. The cells were fixed with PFA (1%) for 10min. After three washing steps, unspecific binding was blocked with BSA (3%, 1 h). Cells were immunostained using anti-CD14 antibody (15µg/mL, Thermo Fisher Scientific) for 12 h, followed by incubation with Alexa594-labeled secondary antibody (Thermo Fisher Scientific). The cells were washed and mounted with ProLong™ Diamond Antifade Mountant with DAPI (Thermo Fisher Scientific). For visualization of ingested 6-FAM-labeled PFOB-NE, cells were analyzed by confocal microscopy (LSM700, Carl Zeiss Microscopy GmbH, Jena, Germany) using ZEN software (Version 2.3, Carl Zeiss Microscopy GmbH, Jena, Germany). 

### 4.8. Statistical Analysis

Data are presented as mean value ± SD. Differences in MFI over the time were analyzed using a two way repeated measures analysis of variance (ANOVA). To analyze monocyte migration or the effect of CD11b- or Fc-inhibition a one way ANOVA was performed. Turkey’s multiple comparisons test was used as a post-hoc analysis to reveal statistical differences between the groups. A paired t-test was performed to analyze the percentage of different monocyte subsets before and after PFOB exposure. Statistical analysis was done using GraphPad Prism© (Version 6.01, GraphPad Software Inc., San Diego, CA, USA). Statistical significance was assumed for *p* < 0.05. 

## 5. Conclusions

PFOB-NE is ingested by human monocytes via active phagocytosis in a time- and subset-dependent manner. Thereby, cell function (phagocytosis, migration) and viability is preserved. PFOB-NE uptake is not significantly altered by the acute inflammatory reaction at day 3 after STEMI.

## Figures and Tables

**Table 1:** Basic properties of the applied PFOB-NE.

Parameter	Value (± SD)
Particle size	209.2 ± 17.4 nm
Zeta-Potential	−45.4 ± 7.8 mv
Polydispersity Index	0.22
Phospholipid type	E 80 S
PFOB fraction	50.5% [*w*/*w*]
Buffer fraction	45.2% [*w*/*w*]
Lipoid fraction	2.9% [*w*/*w*]
Stabilizer fraction	1.4% [*w*/*w*]
^19^F molarity	65.69 mol/L

## Supplementary Materials

The following are available online at https://www.mdpi.com/1420-3049/24/11/2058/s1, Figure S1: Monocyte Gating Strategy, Figure S2: Neutrophils gating strategy, Figure S3: Lymphocyte Gating Strategy, Figure S4: Apoptosis/Necrosis Gating Strategy, Table S1: Inclusion and exclusion criteria, Table S2: Patients characteristics, Table S3: Univariate correlation analysis of several clinical parameters to 6-FAM MFI after 160 min of PFOB-NE challenge.
