# Supplementary material for: Phagocytosis of a PFOB-Nanoemulsion for 19F Magnetic Resonance Imaging: First Results in Monocytes of Patients with Stable Coronary Artery Disease and ST-Elevation Myocardial Infarction
Source: Molecules. 2019 May 30;24(11):2058. doi: 10.3390/molecules24112058 (PMC6600522; doi:10.3390/molecules24112058)
Supplement: Supplementary file 1 [file molecules-24-02058-s001.pdf]

Supplementary materials

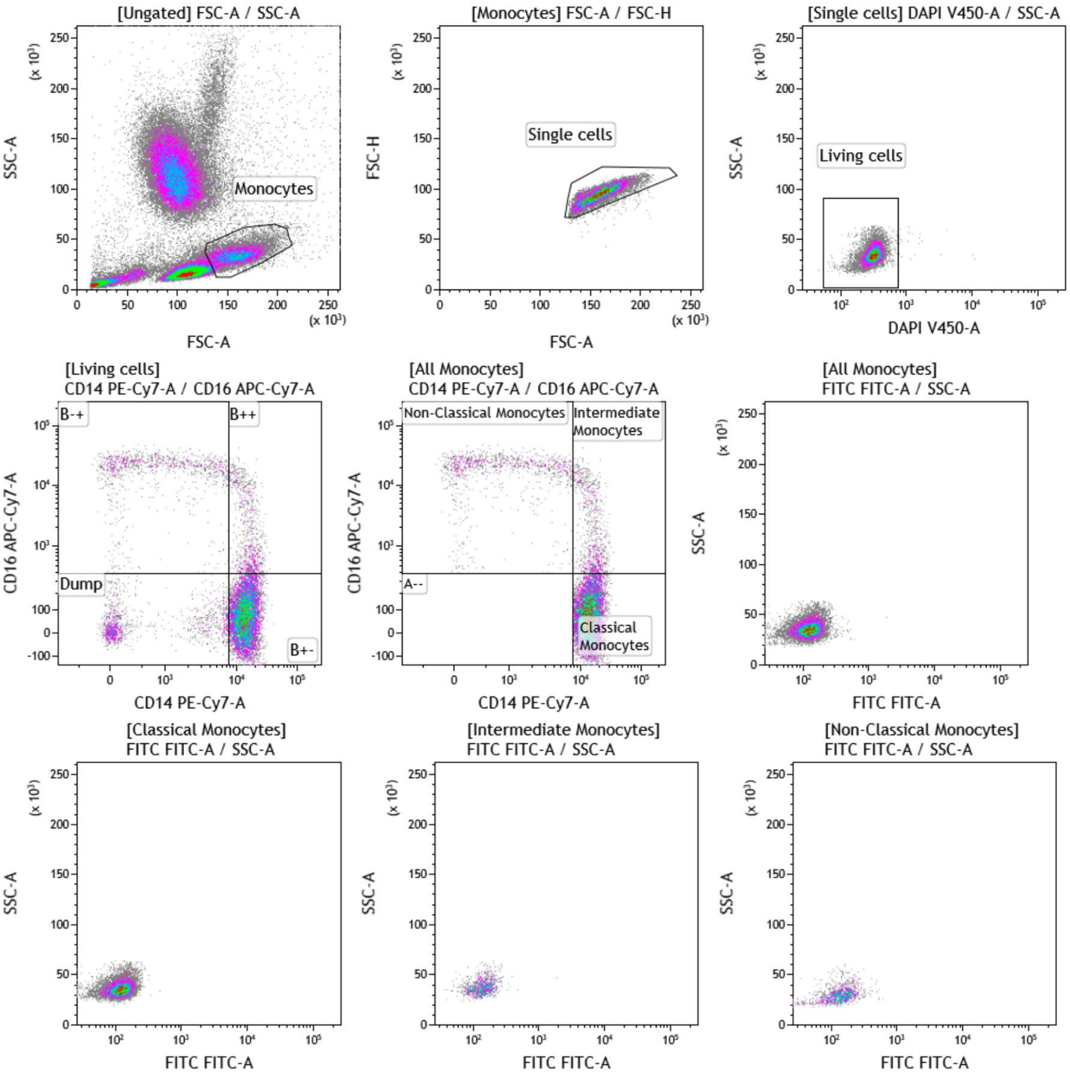

Supplementary Figure S1. Monocyte gating strategy. Monocytes were identified by size and granularity (FSC and SSC). After exclusion of dead cells (DAPI) and cell duplicates (FSC-H, FSC-A) monocyte subsets were identified by CD14 and CD16 staining. 6-FAM fluorescence intensity was measured over the time for all monocyte subsets.

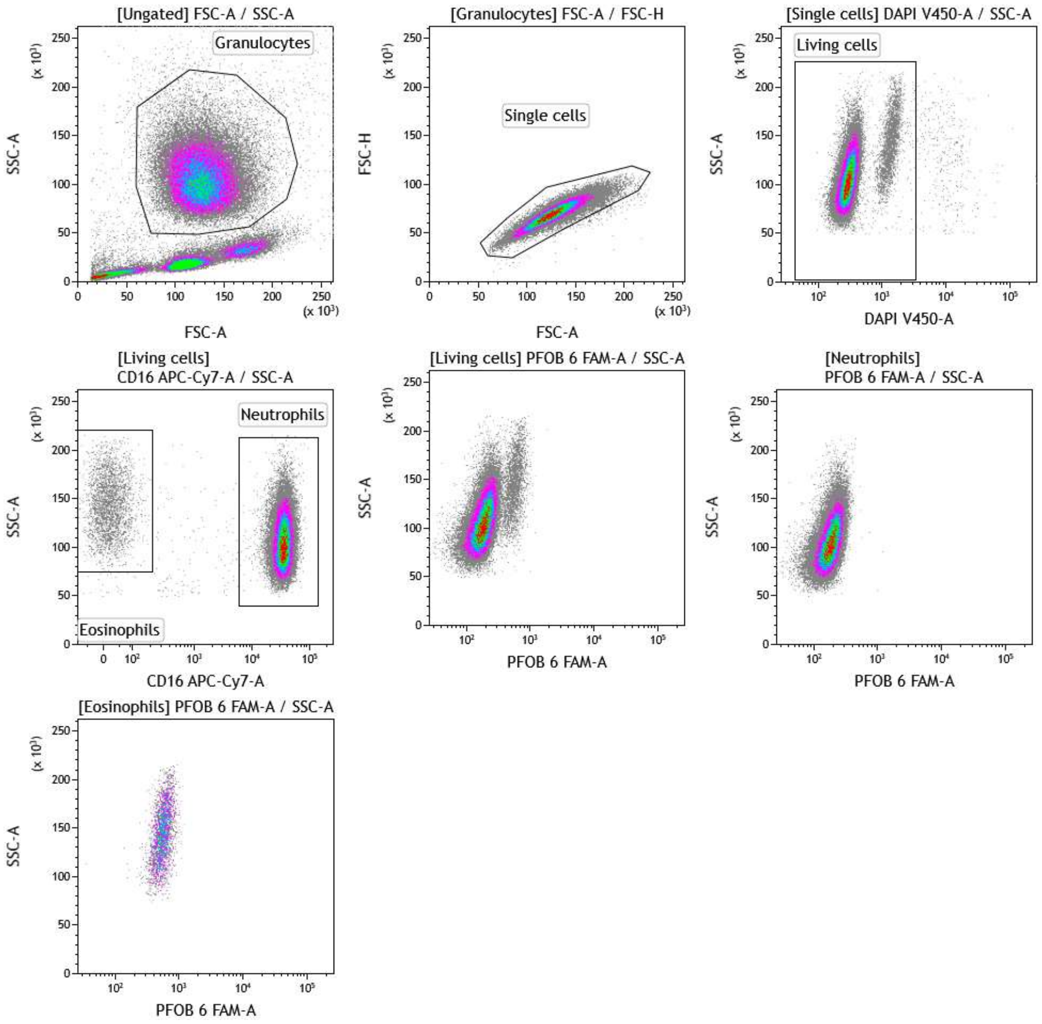

Supplementary Figure S2. Neutrophils gating strategy. Granulocytes were identified by size and granularity (FSC and SSC). After exclusion of dead cells (DAPI) and cell duplicates (FSC-H, FSC-A) neutrophils were identified by CD16 staining. 6-FAM fluorescence intensity was measured over the time.

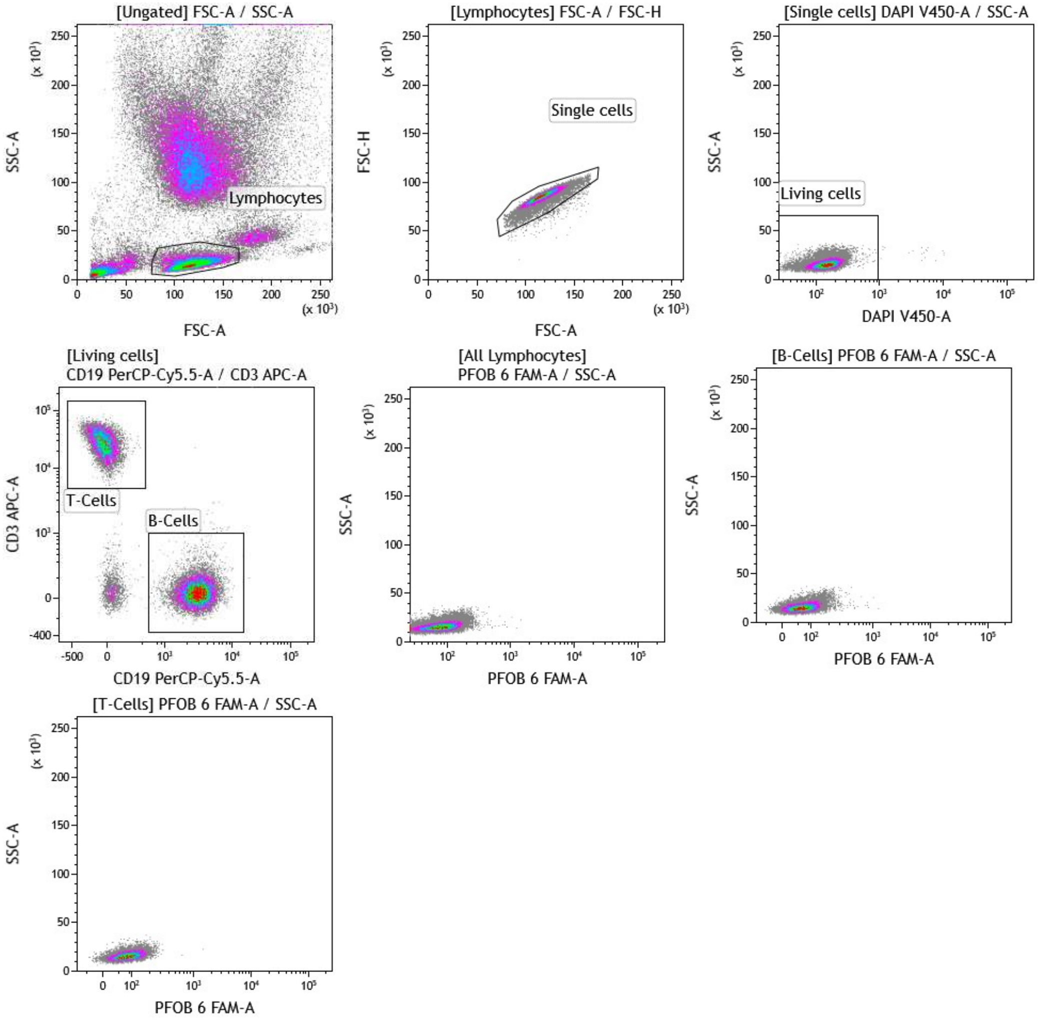

Supplementary Figure S3. Lymphocyte gating strategy. Lymphocytes were identified by size and granularity (FSC and SSC). After exclusion of dead cells (DAPI) and cell duplicates (FSC-H, FSC-A) B cells and T cells were identified by CD19 and CD3 staining. 6-FAM fluorescence intensity was measured over the time.

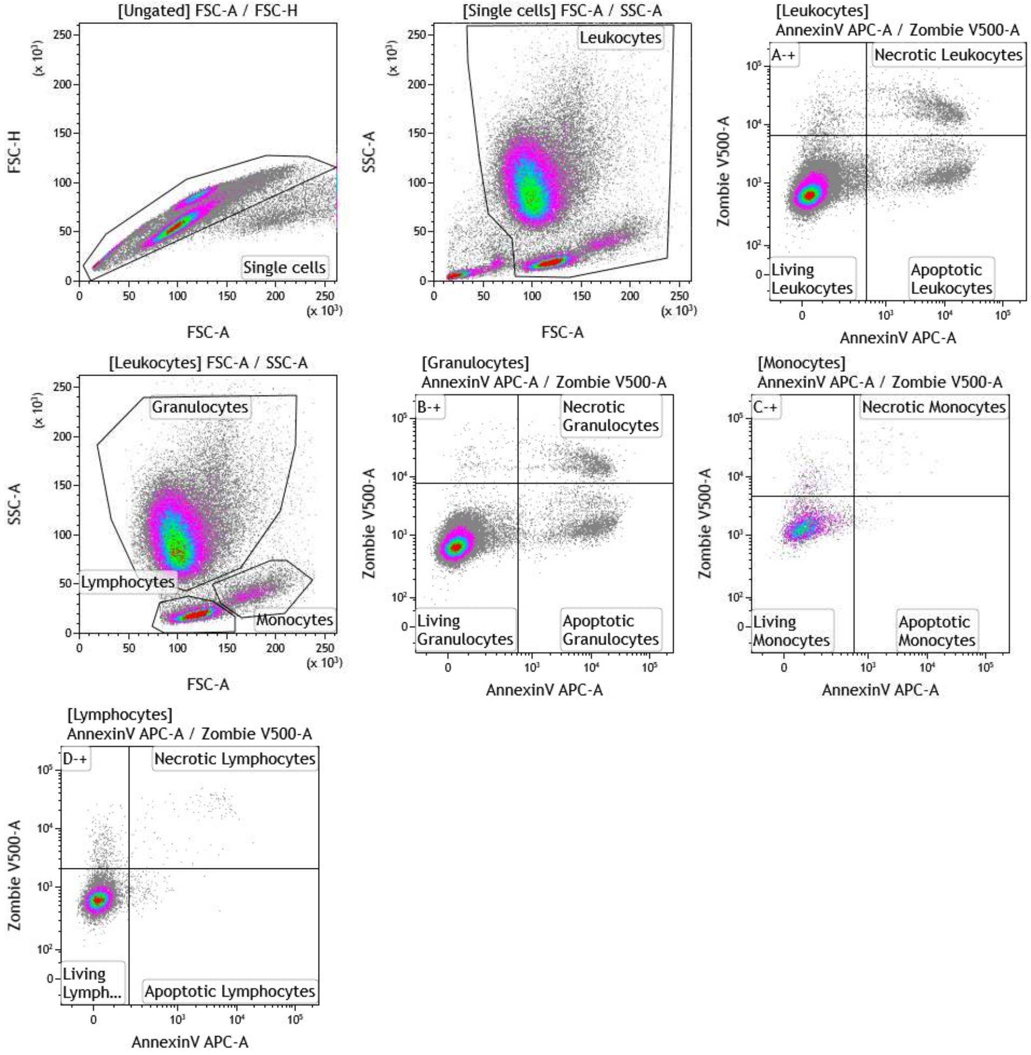

Supplementary Figure S4. Apoptosis/necrosis gating strategy. Leukocytes as well as leukocyte subsets (granulocytes, monocytes, lymphocytes) were identified by size and granularity (FSC and SSC). Cell duplicates were excluded (FSC-H, FSC-A). Apoptosis and necrosis was detected by Annexin V and Zombie staining.

Supplementary table S1. Inclusion and exclusion criteria

Patients with STEMI	
**Inclusion criteria**	**Exclusion criteria**
Diagnosis of STEMI according to the ESC guidelines	Acute infectious disease (Leukocytosis and/or CRP > 10 mg/dl and proven infect focus)
Day 3 after reperfusion	COPD GOLD ≥ III
Age > 40 years	Renal insufficiency, CKD-Stadium ≥ 4
Informed consent	Haematological and/or oncological diseases
	Allergic asthma
	Autoimmune diseases
	Operation within the last 3 months
**Patients with SCAD**	
**Inclusion criteria**	**Exclusion criteria**
Angiographically proven coronary artery disease	Acute infectious disease (Leukocytosis and/or CRP > 10 mg/dl and proven infect focus)
Age > 40 years	Evidence of acute Myocardial infarction up to < 3 months ago
Informed consent	Unstable angina pectoris
	COPD GOLD ≥ III
	Renal insufficiency, CKD-Stadium ≥ 4
	Haematological and/or oncological diseases
	Allergic asthma
	Autoimmune diseases
	Operation within the last 3 months

COPD = chronic obstructive pulmonary disease, CKD = chronic kidney disease, CRP = C reactive protein

Supplementary table S2. Patient characteristics

**Basic characteristics**	**STEMI**	**SCAD**
Number of patients	16	20
Sex (m:f)	13:3	17:3
Age (years)	60.3 ± 8.4	69.2 ± 10.6
BMI (kg/cm2)	29.4 ± 5.2	27.6 ± 3.9
**Cardiovascular risk factors**	**STEMI**	**SCAD**
Arterial Hypertension	62.2 %	95 %
Hyperlipoproteinaemia	31.25 %	60 %
Diabetes mellitus	25 %	35 %
Nicotine abuse	56.25 %	30 %
Positive family history	25 %	10 %
**Laboratory parameters**	**STEMI**	**SCAD**
CK max. (U/l)	887.1 ± 709.8	101.1 ± 44.1
Troponin max. (ng/l)	3259.7 ± 2120.6	22.7 ± 13.5
Leukocytes/µl	9.05 ± 2.8	7.73 ± 1.41
CRP (mg/dl)	4.1 ± 8.1	0.6 ± 0.9
Creatinine (mg/dl)	1.1 ± 0.3	0.9 ± 0.2
eGFR (ml/min)	74.0 ± 19.3	79.3 ± 13.3
Percent Monocytes of all Leukocytes (%)	4.9 ± 1.4	4.9 ± 1.29

BMI = body mass index, CK = creatine kinase, CRP = C reactive protein, eGFR = estimated glomerular filtration rate

Supplementary table S3. Univariate correlation analysis of several clinical parameters to 6-FAM MFI after 160 min of PFOB-NE challenge.

	STEMI			SCAD		
**Parameter**	**r**	**r^2**	**p-value**	**r**	**r^2**	**p-value**
CMR infarct size	-0,02860	0,0008182	0,9515	n.a.	n.a.	n.a.
CK max	0,07617	0,005801	0,7792	n.a.	n.a.	n.a.
CRP	-0,2551	0,06505	0,3589	-0,1569	0,02461	0,5213
Age	-0,2726	0,07431	0,3070	0,1042	0,01086	0,6619
Hemoglobin	0,1615	0,02609	0,5501	0,1436	0,02062	0,5459
eGFR	-0,3639	0,1325	0,1658	-0,1208	0,01458	0,6120
Creatinin	0,4452	0,1982	0,0840	-0,03001	0,0009006	0,9001
Leukocyte count	-0,2675	0,07154	0,3166	0,2778	0,07720	0,1993
Monocyte count	0,1119	0,01251	0,6800	0,2969	0,08817	0,2036

CMR = cardiac magnetic resonance imaging, CK = creatine kinase, CRP = C reactive protein, eGFR = estimated glomerular filtration rate
